# Contrasting cannabinoid receptor 2 (CB2R)-mediated responses in two different models of Blood Brain Barrier in the context of HIV

**DOI:** 10.21203/rs.3.rs-8310572/v1

**Published:** 2025-12-09

**Authors:** Violaine Delorme-Walker, Kaylin Au, Wei Ling Lim, Takayo Sasaki, Tomomi Furihata, Daniel Siqueira Lima, Jennifer Iudicello, Richard Milner, Maria Cecilia Garibaldi Marcondes

**Affiliations:** San Diego Biomedical Research Institute, San Diego, CA, 92121; San Diego Biomedical Research Institute, San Diego, CA, 92121; San Diego Biomedical Research Institute, San Diego, CA, 92121; San Diego Biomedical Research Institute, San Diego, CA, 92121; Department of Clinical Pharmacy & Experimental Therapeutics, School of Pharmacy, Tokyo University of Pharmacy and Life Sciences, Tokyo 192-0392, Japan; San Diego Biomedical Research Institute, San Diego, CA, 92121, HIV Neurobehavioral Research Center, University of California San Diego, CA, 92103; Department of Psychiatry, HIV Neurobehavioral Research Center, University of California San Diego, CA, 92103; San Diego Biomedical Research Institute, San Diego, CA, 92121; San Diego Biomedical Research Institute, San Diego, CA, 92121

**Keywords:** Cannabis, Human Immunodeficiency Virus, Blood-Brain Barrier, Latency

## Abstract

The infection with the Human Immunodeficiency Virus is associated with several comorbidities despite suppressive antiretrovirals, which include consequences to the Central Nervous System (CNS), where disruption of the blood-brain barrier (BBB) a major underlying factor in the resulting chronic inflammation and pathogenesis. Currently, the use of cannabis and cannabinoid derivatives among persons living with HIV (PWH) is common. Despite perceived benefits, we have previously identified context-dependent effects of cannabis use, including in vascular biomarkers. In this study, we used an in vitro multicellular BBB model with two different human stable cerebrovascular endothelial cell lines (hCMEC/D3 and HBMEC/ci18) to test the effects of cannabinoids via their receptors on integrity and function in the context of exposure to conditioned media from HIV latently infected promonocytes. We found that the two cell lines had similar responses to HIV-conditioned media by increasing permeability to dextran and decreasing tight junction proteins. However, their response to cannabinoids, particularly via the cannabinoid receptor 2 (CB2R) was markedly contrasting, with hCMEC/D3 cells showing improvement of BBB integrity by all measures, and HBMEC/ci18 cells showing no benefits or aggravation of damage. While the contrasting effects were not due to differences in viability or proliferation, GPCR response with production of cAMP was above 50-fold higher in hCMEC/D3 cells, including at baseline, in correlation with higher availability of CB2R compared to HBMEC/ci18. Our study suggests that CB2R levels and activation threshold on cerebrovascular endothelium may dictate improvements versus aggravating effects of cannabis to the BBB of PWH.

## Introduction

The management and cure of chronic HIV-1 infection remain a challenge due to the persistence of cellular reservoirs and chronic inflammation, despite antiretroviral therapies (ART)[1–4]. In the brain, where most HIV-1 targets and reservoirs are of myeloid origin[5–7], chronic inflammation contributes to neurological and neurocognitive impairment (NCI)[8–12]. One of the most overlooked consequences of chronic neuroinflammation is vascular injury, which is a risk factor for atherosclerosis and cardiovascular disease (CVD), but also a critical component of cerebrovascular pathology that is linked to NCI [13] especially in persons living with HIV (PWH)[14]. Substance use is also a prevalent HIV comorbidity [15–17] with effects on both CVD and NCI. The mechanisms by which addictive substances and HIV interact are multifactorial and not well understood, with impacts going beyond reward and neurotransmitters[18, 19]. Cerebrovascular endothelial cells and other components of the blood-brain barrier (BBB), as well as myeloid cells that are targets of HIV in the brain such as macrophages and microglia, all express receptors to neurotransmitters and drug compounds[20, 21]. Thus, PWH that are substance users may show a wide range of cellular responses [22–24] that could depend on the type of used drug and use patterns[25].

Among used substances, cannabis is more prevalent among PWH than in the general population, medically and recreationally[26, 27], and used in many forms. Reported benefits include relief from symptoms of HIV and side-effects from its treatments[28–30]. Observational studies report mixed effects of cannabis on disease characteristics such as CD4^+^ T-cells and HIV RNA[31, 32]. Reported benefits include lower pro-inflammatory biomarkers in cells, plasma and cerebrospinal fluid (CSF)[33–35], lower immune activation and fewer CD16^+^ monocytes[34, 36], with improved prognosis[37, 38]. Other studies have suggested that chronic cannabis is linked to vascular disease[39, 40] and risk of cardiovascular complications[41]. For instance, one study on chronic heavy users has shown more CVD events in both PWH and persons without HIV[42]. However, it is possible that the effects of cannabis on reducing inflammation may counterbalance adverse vascular effects.

The two primary constituents of cannabis, ((Δ9-tetrahydrocannabinol [THC], and cannabidiol [CBD]), and their receptors (CBRs), along with the endocannabinoid system (ECS) including key enzymes (FAAH, MAGL), may influence inflammation, cognition, sleep, appetite, pain, and mood[43]. These factors also modify endothelial states associated with vascular integrity and disorders[44–49] that have implications to the brain and NCI, in relation to the BBB. Among the CBRs, CB1R and CB2R, as well as the orphan receptor GPR55, are known to be expressed by endothelial cells[50, 51]. Both major cannabinoid receptors are Gi/o-coupled proteins that inhibit adenylyl cyclase activity, activate voltage-gated calcium channels, initiate mitogen-activated protein kinase (MAPK) and activate phosphoinositide 3-kinase (PI3K)-Akt pathways via induction of cyclic adenosine 3,5 monophosphate (cAMP) [52–54]. CB2R is of particular interest as it is broadly expressed, and downstream effects regulate cell growth and survival, influence endothelial cell functions and modulate immune cells that cause inflammation [55].

Recently, we found that moderate cannabis use may improve NCI[26], and prevent changes in the expression of polygenic components of vascular and leukocyte migration pathways in the context of HIV, but not in uninfected subjects[56]. Cannabis use patterns are linked to differences in the expression of the vascular biomarkers vascular cell adhesion molecule (VCAM)-1, intercellular adhesion molecule (ICAM)-1 and urokinase activator plasminogen receptor (uPAR), that serve as biomarkers to detect and monitor NCI in the context of HIV[57–60]. However, other studies report no differences, or detrimental effects in HIV-negative populations[41], raising the possibility that the observed effects of cannabis, whether beneficial or detrimental, may be largely domain and context-dependent.

Differential consequences of CB2R activation may also derive from genetic variations resulting in molecular and outcome differences[61], including in inflammatory and autoimmune pathogeneses[62]. Levels of CB2R in peripheral blood may also serve as biomarkers of mental health disorders, such as schizophrenia, with a negative correlation between mRNA levels and processing or working memory speed [63].

The identification of CB2R on cerebral endothelial cells suggests that this receptor may play a role in regulating the BBB in correlation with inflammatory processes in the CNS. For instance, CB2R expression is highly upregulated on isolated blood vessels following traumatic brain injury, with a positive impact of its activation for recovery [64]. While in the human population, and especially in the context of HIV infection, overall benefits may be context-dependent[65], it is currently unknown whether activation of cannabinoid receptors on the BBB can directly benefit vascular health and thereby reduce damage occurring in the HIV-infected brain, considering potential individual differences in the expression of cannabinoid receptors.

With this question in mind, here we describe in vitro experiments that seeded from the observation that different cerebrovascular endothelial cell lines respond in opposite manners to exposure to cannabinoid receptor agonists, particularly CB2R, in the context of HIV. By potentially mimicking different vascular responses to cannabinoids in humans, the comparison of these two human cerebrovascular cell lines using an in vitro system facilitated the exploration of molecular mechanisms that may explain context-dependent effects or individual differences in vascular outcomes influencing BBB functions that underlie inflammation in the brain. Our findings indicate that cannabinoid receptor signaling contributes to both beneficial and harmful effects that could explain context-dependent outcomes in PWH that are cannabis users. These findings could form the molecular basis of novel therapeutic strategies to improve outcomes in PWH who develop NCI, and biomarkers that can predict individuals that can mostly benefit from these approaches.

## Material and Methods

### BBB Cell cultures

#### Human Cerebrovascular Endothelial cells, Astrocytes and Pericytes

The immortalized hCMEC/D3 cell line was donated by Dr. Vadivel Ganapathy (Texas Tech University Health Sciences Center). hCMEC/D3 cells (passage 14–24) were seeded on 0.1% gelatin-coated (Embryomax, Millipore, Burlington, MA, USA) culture dish in EndoGRO basal medium with EndoGRO-MV-VEGF supplement kit consisting of 5 ng/ml rhVEGF, 5 ng/ml rhEGF, 5 ng/ml rhFGF, 15 ng/ml rhIGF-1, 10 mM Glutamine, 1 μg/ml Hydrocortisone hemisuccinate, 0.75 U/ml Heparin sulfate, 50 μg/ml Ascorbic acid and 5% FBS (Millipore). Cells were maintained at 37°C in a humidified atmosphere (5% CO_2_/95% air). Medium was changed every 3–4 days until cells reached confluence. Conditionally immortalized human brain microvascular endothelial cells clone 18 (HBMEC/ci18), human brain pericytes clone 37 (HBPC/ci37), and human astrocytes clone 35 (HASTR/ci35) were established, validated and kindly donated by Pr. Tomomi Furihata (Tokyo University of Pharmacy and Life Sciences, Japan). All cells were seeded on culture dishes coated with 50 μg/ml Cultrex 3-D Culture matrix rat Collagen I (R&D Systems, Minneapolis, MN). For maintenance, HBMEC/ci18 cultures were grown in the same medium as for hCMEC/D3. HBPC/ci37 cells were maintained in complete pericyte growth medium (PGM) consisting of basal medium, 2% FBS, 1% pericyte growth supplement and 1% penicillin/streptomycin (ScienCell, Carlsbad, CA). HASTR/ci35 cells were grown in complete astrocyte growth medium (AGM) consisting of DMEM + GlutaMAX, 10% FBS and 1% N2-supplement (Gibco, ThermoFisher Scientific, Waltham, MA). All culture media for the conditionally immortalized human cells contained 4 μg/ml blasticidin S (Tocris, Minneapolis, MN). HBMEC/ci18, HASTR/ci35 and HBPC/ci37 cell lines were maintained at 33°C for growth and at 37°C for differentiation, in a humidified atmosphere (5% CO_2_/95% air). Medium was changed every 3–4 days until cells reached confluence.

#### Human promonocytes

Human promonocytes uninfected (U937) and U937-derived latently infected with HIV-1 (U1) were maintained using RPMI-1640 medium (Gibco, Thermofisher, Waltham, MA, USA) supplemented with 10% fetal bovine serum (Hyclone, Cytiva, Marlborough, MA, USA), 2 mM Glutamine (Gibco) and 100 U/ml penicillin/streptomycin (Gibco). Cultures were maintained in 75 cm^2^ flasks (Genesee Scientific, San Diego, CA, USA) at 37°C in a humidified atmosphere (5% CO_2_/95% air).

### BBB co-culture system

Transwell inserts (clear polyester (PET) membranes, 6.5 mm diameter, 0.4 μm pores, #3470, Corning, Corning, NY, USA) in 24-well plates were coated with a combination of 50 μg/ml Cultrex 3D culture matrix rat collagen I (R&D systems, Minneapolis, MN, USA), 50 μg/ml Human Collagen IV (Advanced BioMatrix # 5022) and 50 μg/ml fibronectin (#F1141, Sigma) for 2 h at 37°C. HBPC/ci37 pericytes were seeded on the underside of the transwells at a density of 2.7 × 10^4^ cells/cm^2^. HASTR/ci35 astrocytes were seeded on the bottom of the 24-well plate at a density of 2.5 × 10^4^ cells/cm^2^. After 24 hours, the pericyte and astrocyte media were replaced with corresponding media without FBS and blasticidin S. The astrocyte medium was replaced with astrocyte medium without FBS and blasticidin S, supplemented with 1 mM dibutyryl-cAMP (Selleckchem). Cells were allowed to differentiate for 24 hours at 37°C. Endothelial cells were then seeded on the apical side of the transwell at 10^5^ cells/cm^2^ or 1.3 × 10^5^ cells/cm^2^ for hCMEC/D3 and HBMEC/ci18, respectively. The transwell inserts with endothelial cells and pericytes were transferred into the 24-well plates containing the astrocytes. Cells were grown in EndoGRO culture medium mentioned above, VEGF and EGF free, in the apical chamber, and Neurobasal medium (Life Technologies) containing 1% N2-supplement (ThermoFisher Scientific) and 2 mM Glutamine (Gibco) in the lower chamber. To control the establishment of a functional BBB, each endothelial cell line, hCMEC/D3 and HBMEC/ci18, were also seeded as monocultures in transwells. BBB co-cultures or endothelial cell monocultures were grown for 5 days. Endothelial monolayer integrity was checked by permeability measurements on day 5 and then on day 6 after exposure to conditioned medium or treatments. In each experiment, one transwell was used without cells as control for insert permeability.

### HIV Conditioned medium and p24 levels

U937 and U1 cells were cultivated at the density of 10^6^ cells/ml in 75 cm^2^ flasks in EndoGRO medium without VEGF and EGF. High levels of virus expression were induced in U1 cells by stimulation with latency reversal agents: 10 nM and 1 μM PEP005 (Tocris, Minneapolis, MN, USA), 1 μM iBET151 (Sigma Aldrich, Burlington, MA, USA) or 10 ng/ml and 100 ng/ml PMA (Sigma Aldrich, Burlington, MA, USA) for 48 h. Culture supernatants were harvested by centrifugation after 5 days (U937 and U1) or after 48 h in presence of latency reversal agents. Conditioned media were stored at −20°C until used in assays. Levels of p24 antigen in U1 conditioned media were determined by ELISA (HIV-1 p24 ELISA assay, Xpress Bio, Frederick, MD, USA), according to the manufacturer’s instructions.

### Vascular inflammation and network analysis

To characterize the contents of the conditioned media, U937 and U1 supernatants were analyzed using LEGENDplex^™^ Human Vascular Inflammation (BioLegend, San Diego, CA, USA) multiplex bead-based assay Panels 1 and 2, following the manufacturer’s instructions. After completion of the reaction, the samples were acquired on a CytoFLEX S flow cytometer (Beckman Coulter, Brea, CA, USA) and the results were analyzed using LEGENDplex^™^ Data Analysis Software Version 2025-05-01 (BioLegend). The concentration of each analyte was determined using a standard curve generated in the same assay. All conditions were tested in triplicate, and the assays were repeated at least four times. Network analysis and visualization was performed using Cytoscape v3.10.2 software[66, 67] with ReactomeFIPlugIn[68].

### Treatments

Endothelial cell monolayers seeded on transwell inserts, glass coverslips or cell culture plates for 5 days were exposed to control conditioned medium (U937) or HIV-conditioned medium (U1) for 24 h at 37°C for hCMEC/D3 or 33°C for HBMEC/ci18. Cells were pre-treated with DMSO (0.1% v/v) (ATCC, Frederick, MD, USA) as control, or with 0.1 μM, 1 μM or 10 μM cannabinoid receptor agonists or antagonists as follow: ACEA and AVE1625 for CB1R, HU-308 and AM630 for CB2R, O-1602 and ML 193 for GPR55, respectively (all from Tocris, Minneapolis, MN, USA). Cells were pre-treated for 30 min in EndoGRO VEGF, EGF-free medium with DMSO (0.1% v/v), cannabinoid receptor agonists or antagonists, prior to 24 h incubation with conditioned medium supplemented with the same compounds. Treatment with 10 ng/ml TNFα (Peprotech, Cranbury, NJ, USA) was used as a positive control for endothelial cell monolayer damage.

Endothelial monolayers were also incubated for 24 h with 0.01 μM, 0.1 μM, 1 μM or 10 μM Cannabidiol (CBD, PhytoLab, Millipore Sigma, Burlington, MA, USA) or (−)-trans-Δ^9^-tetrahydrocannabinol (THC, RTI International, Research Triangle Park, NC, USA), alone or together, in combination with control conditioned medium (U937) or HIV-conditioned medium (U1). Methanol and ethanol were used as vehicle controls to THC and CBD, respectively.

### Measurement of paracellular permeability

Effects of HIV-conditioned media and CB2R signaling on BBB integrity were assessed by measuring paracellular permeability to three fluorescently-labeled dextrans: 4 kDa FITC-conjugated dextran (Milllipore-Sigma), 10 kDa Cascade Blue-conjugated dextran (Invitrogen) and 40 kDa Texas Red-conjugated dextran (Invitrogen). Briefly, hCMEC/D3 and HBMEC/ci18-containing inserts were washed with Hank’s balanced salt solution (HBSS) + 10 mM Hepes pH 7.5. Inserts were then incubated with 700 μl HBSS + 10 mM Hepes pH 7.5 in the basolateral side and 300 μl HBSS + 10 mM Hepes pH 7.5 containing 10 μg/ml of each fluorescently labeled-dextran onto the apical side. Inserts were incubated 1 h at 37°C for hCMEC/D3 or 33°C for HBMEC/ci18. The medium from the basolateral well was collected and fluorescence intensity was measured in triplicate on a VersaMax Spectrophotometer using the following wavelengths: Ex405/Em440 for the Cascade Blue-conjugated dextran, Ex485/Em525 for the FITC-conjugated dextran and Ex560/Em615 for the Texas Red-conjugated dextran. The concentration of the corresponding dextrans were determined and the permeability coefficient values were calculated using the following equation:

P=(Vr/C0)×(1/S)×(C1/t),

Where P is the apparent permeability, Vr is the volume of medium in the basolateral side of the chamber (Vr = 0.7 cm^3^), C0 is the concentration of the fluorescent dextran in the apical side of the transwell at t0 (C0 = 10 μg/ml), S is the surface area of the monolayer (S = 0.33 cm^2^), C1 is the concentration of the fluorescent dextran in the basolateral side of the chamber after incubation and t is the incubation time (t = 3600 s).

### Cell viability

Endothelial cells were seeded at the density of 10^5^ cells/cm^2^ in 24-well plates coated with 0.1% gelatin (hCMEC/D3) or 50 μg/ml Cultrex 3-D Culture matrix rat Collagen I (HBMEC/ci18). Cells were cultured in EndoGRO medium without VEGF and EGF for 5 days before being incubated for 24 h in presence of conditioned media, or in cell culture medium with 0.1% DMSO (v/v), 0.1 μM, 1 μM or 10 μM HU-308. Each culture condition was tested in triplicate. Three wells without cells were used as blank control. Cell viability was measured with CyQUANT^™^ XTT Cell Viability Assay (ThermoFisher Scientific), according to the manufacturer’s instructions. XTT reagent was incubated with the cells for 4 h at 37°C or 33°C for hCMEC/D3 and HBMEC/ci18 cells, respectively. Absorbances at 450 nm (XTT specific) and at 660 nm (background signal) were measured on a VersaMax Spectrophotometer. Absorbance was quantified as follow: Absorbance = [Abs 450 nm (sample) – Abs 450 nm (blank no cells)] - Abs 660 nm (sample). The final absorbance was normalized to U937 control conditioned medium for experiments with U1, or to cell culture medium with 0.1% DMSO for experiments with HU-308.

### Cell proliferation

hCMEC/D3 and HBMEC/ci18 cells were seeded at the density of 10^5^ cells/cm^2^ in 12-well plates containing glass coverslips (#1.5, Carolina, Burlington, NC) pre-coated with 50 μg/ml Cultrex 3D culture matrix rat collagen I (R&D systems). Cells were cultured in EndoGRO medium without VEGF and EGF for 5 days before being incubated for 24 h in presence of conditioned media, with 0.1% DMSO (v/v) or 10 μM HU-308, in combination with 10 μM 5-Bromo-2’-deoxy-Uridine (BrdU). Immunofluorescence detection of BrdU was performed using BrdU labelling and detection kit I (Roche molecular biochemicals, Indianapolis, IN), according to the manufacturer’s instructions. Cells were co-stained with DAPI to detect nuclei. Coverslips were mounted on slide with Prolong Gold Antifade (Invitrogen). Images were acquired using a 20x objective on an Axioskop2 plus microscope (Carl Zeiss, Dublin, CA, USA) equipped with an Infinity 3S camera (Lumenera, Ottawa, Ontario, Canada) and Infinity Analyze imaging software (Lumenera). Image analysis and quantification of the percentage of BrdU positive cells over total cells were performed with ImageJ/FIJI software (NIH).

### Detection of Cyclic AMP

hCMEC/D3 and HBMEC/ci18 endothelial cells were seeded on a 96-well plate at 10^5^ cells/cm^2^ until forming a monolayer. Following 24 h of treatments as described above, the cells were incubated with cAMP assay buffer (HBSS 1x, 10 mM Hepes and 30 μM of Forskolin). Cyclic AMP was measured using the HitHunter cAMP assay for Biologics kit (DiscoverX Corporation, Fremont, CA) following manufacturer’s protocols. Chemiluminescence signals were measured on a standard luminometer at 0.5 sec/well. All conditions were tested in duplicate and assays were performed in triplicate.

### RT-PCR

hCMEC/D3 and HBMEC/ci18 cells were seeded at the density of 1.5 × 10^5^ cells/well on 12-well plates coated with 0.1% gelatin (Millipore) or 50 μg/ml Cultrex 3D culture matrix rat collagen I, respectively. Cells were cultured in EndoGRO medium without VEGF and EGF for 5 days before being incubated for 24 h in presence of conditioned media, with 0.1% DMSO (v/v) or 10 μM HU-308. Total RNA was extracted from samples using Nucleospin RNA isolation kit (Macherey-Nagel, Allentown, PA) and cDNA was obtained using RT^2^ First-strand kit (Qiagen), according to the manufacturers’ instructions. SYBR Green real-time PCR was performed using the RT^2^ PCR Primer set for human CB1 receptor (CNR1, GenGlobe ID: PPH01504A-200, Qiagen), CB2 receptor (CNR2, GenGlobe ID: PPH02723A-200, Qiagen) and GPR55 (GenGlobe ID: PPH11293B-200, Qiagen) primers. Human GAPDH (GenGlobe ID: PPH00150F-200, Qiagen) was used as housekeeping control. The expression was normalized to mRNA level of the housekeeping gene GAPDH and to mRNA level in control conditioned medium (U937). The relative mRNA expression was determined by measuring 2^−ΔΔCt^ values.

### TJ immunofluorescence and imaging

After measurement of endothelial cell permeability, transwells were washed three times in calcium- and magnesium-free phosphate buffered saline (PBS). Cells were then fixed in cold methanol/acetone (50%/50%) for 20 minutes at −20°C. After rinsing in PBS, cells were incubated with blocking solution consisting of PBS supplemented with 5% FBS and 0.3% Triton X-100. Endothelial cells on the apical side of the transwell were incubated with primary antibodies against Zonula Occludens-1 (ZO-1, Invitrogen #61–7300), Occludin (Invitrogen #33–1500) or CD31 (BD Pharmingen #555025). Pericytes on the underside of the transwell membrane were incubated with antibodies against NG2 Chondoritin Sulfate (Sigma #AB5320). Astrocytes on the bottom of transwells were labeled with antibodies against Glial Fibrillary Acidic Protein (GFAP, Sigma #MAB360) or Glutamine Synthetase (GS, Sigma # G2781). All antibodies were diluted 1:100 in PBS supplemented with 2% BSA and 0.1% Tx-100. Transwells were incubated overnight at 4°C in a humidified chamber. Following rinses in PBS, cells were incubated for 3 h with secondary antibodies donkey anti-rabbit AlexaFluor488 or anti-mouse AlexaFluor647 (dilution 1:500, ThermoFisher Scientific) in combination with DAPI (dilution 1:1000, ThermoFisher Scientific) to stain nuclei. All antibodies were diluted in PBS supplemented with 2% BSA and 0.1% Triton X-100. Insert membranes were cut out with a scalpel, mounted on a glass slide with Prolong Gold Antifade and coated with glass coverslip.

Fluorescent images were acquired either on an Axioskop2 plus microscope (Carl Zeiss, Dublin, CA, USA) equipped with an Infinity 3S camera (Lumenera) controlled by Infinity Analyze imaging software (Lumenera) by using a 40x objective or on a spinning disk confocal (Yokogawa, PerkinElmer) confocal microscope Nikon TE 2000-U (Nikon) equipped with a CoolSnapHQ camera (Photometrics) using a 40x/1.3 NA Plan Fluor objective lens (Nikon). The intensity of the entire area of fluorescence images was considered for analysis. The percentage of cells with altered junctions was measured by counting the number of cells with discontinued ZO-1 labelling around the cell, over the total number of cells. All quantifications were performed using ImageJ/FIJI (NIH).

### Statistical analysis

Data are expressed as mean ± standard error of mean (SEM). Each experiment was performed in triplicate. Unpaired, two-tailed Student’s *t*-test was used to evaluate significant differences between two groups. Analysis of Variance followed by Bonferroni’s multiple comparisons were used for condition and cell line comparisons when applicable. *P* values < 0.05 were considered statistically significant.

## Results

**Figure 1:**
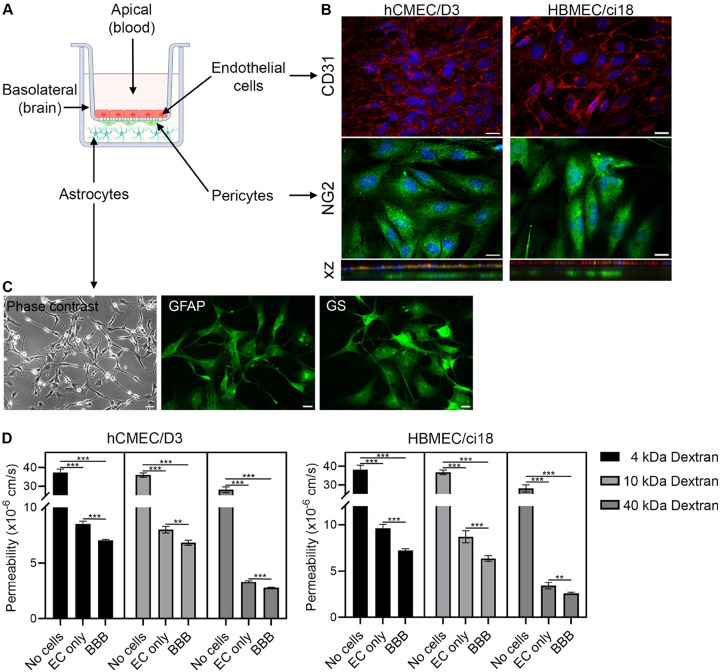
Construction of a multicellular human BBB system. A) Scheme of the human BBB model containing endothelial cells (hCMEC/D3 or HBMEC/ci18), and supporting cells, astrocytes (HASTR/ci35) and pericytes (HBPC/ci37), and indication of the blood and brain compartments. B) Immunofluorescence indicating CD31 endothelial cell monolayers and NG2+ pericytes. Scale bar, 20 μm. Bottom panels are z projections showing the 3D coexistence and interaction between cellular types in the model. C) Detection of astrocytes in the basolateral compartment, visualized by phase contrast and confirmed by immunostaining for glial fibrillary acidic protein (GFAP) and glutamine synthase (GS). Scale bar, 20 μm. D) Permeability to dextran particles (4, 10 and 40kDa) in membranes with no cells (control), with endothelial cells alone (EC only, hCMEC/D3 or HBMEC/ci18), and with the complete multicellular BBB system. Statistical significance is indicated by lines. **p<0.01, ***p<0.0001.

We have developed an in vitro multicellular BBB model (Fig. 1A) using cerebrovascular endothelial cells (CD31-positive), pericytes (NG2-positive, Fig. 1B) and astrocytes (GFAP-positive, Fig. 1C), which developed effective physical interactions detectable on stack images and establishing a functionally tight barrier to large molecules such as dextran (4, 10 and 40 kDa), especially when compared to endothelial cell (EC) monocultures (Fig. 1D). The establishment of functional BBBs was successful using either one of two different cerebrovascular endothelial cell lines, hCMEC/D3 and HBMEC/ci18 (Fig. 1D).

**Figure 2:**
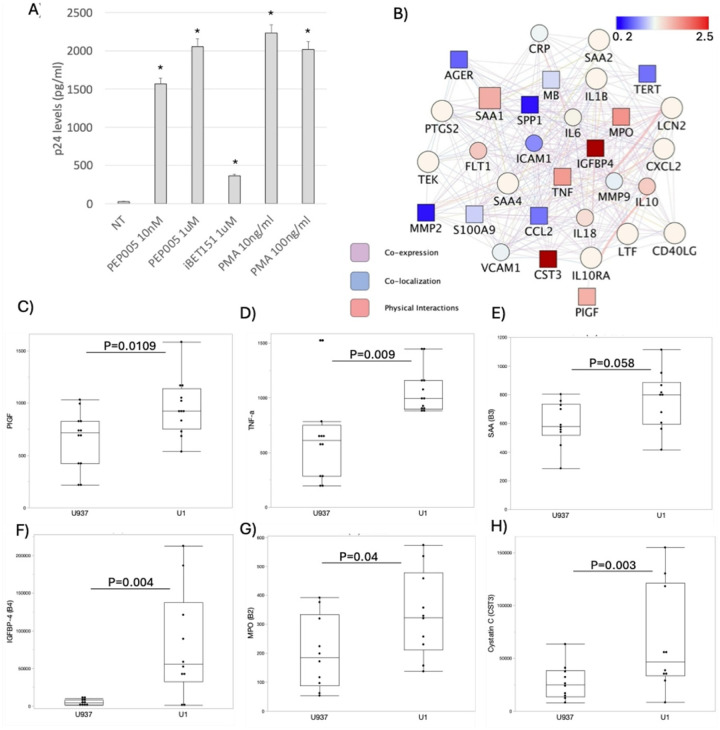
Confirmation of latency and characterization of the conditioned media. **A)** Levels of p24 measured by ELISA in supernatants from U1 cells that were not treated (NT), or treated with latency reversal agents PEP005, iBET151 and PMA at indicated concentrations. **B)** Network analysis of the conditioned media proteome measured by flow cytometry-based assays, showing the fold change comparison of vascular inflammation molecules between U1 and U937 conditioned media, where red shades indicate upregulation and blue shades indicate downregulation in U1 cells compared to U937 conditioned media. Squares indicate significant p values. **(C-H)** Protein levels in U937 and U1 conditioned media in pg/ml, normalized to standard curve. Dots indicate individual measurements, and expressed in mean ± SEM. **C)** PIGF, **D)** TNFa, **E)** SAA, **F)** IGFBP-4, **G)** MPO and **H)** Cystatin C. P values are indicated.

These multi-cells systems were used to examine the impact of conditions that replicate the HIV-infected brain in the ART era, specifically how an environment containing latently infected myeloid cells affect BBB. The latently infected environment was emulated by the incubation of BBB systems with conditioned media obtained from cultures of U1 promonocytes latently infected with HIV1, which have been derived from parent U937 promonocytes [69]. Thus, a control environment was created with conditioned media from uninfected parent cells (U937). The characterization of the U1 conditioned media collected 48 h after cell split, and performed in 6 independent measures in duplicate, revealed a low level p24 average of 38.41 pg of p24/ml (± 3.33). The latent state of the U1 cells was confirmed by the incubation with latency reversal agents PEP005, iBET and PMA, which significantly enhanced HIV transcription, as revealed by p24 levels (Fig. 2A). The supernatants were further characterized by flow cytometry-based proteome assays, indicating differences in levels of a network of vasoactive and pro-inflammatory proteins between uninfected U937 and latent U1 cells (Fig. 2B). For instance, U1 conditioned media contained increased levels of Placental Growth Factor (PIGF) (Fig. 2C), tumor necrosis factor alpha (TNFa) (Fig. 2D), insulin-like growth factor binding protein-4 (IGFBP4) (Fig. 2F), myeloperoxidase (MPO) (Fig. 2G), and Cystatin C (CST3) (Fig. 2H). Serum Amyloid A (SAA) (Fig. 2E) was also increased at a substantial level. On the other hand, inflammatory proteins such as the receptor for advanced glycation end products (RAGE), Osteopontin (SPP1) and CCL2 were significantly lower in U1 conditioned media compared to control U937 supernatants (p = 0.004, 0.05, 0.05, respectively) in agreement with the latency phenotype. The increase of proteins with described effects on the BBB suggests that the model is adequate to mimic the endothelial damage occurring in the brain environment in the context of latent HIV, and to test how cannabinoid signaling may interfere with those effects.

**Figure 3:**
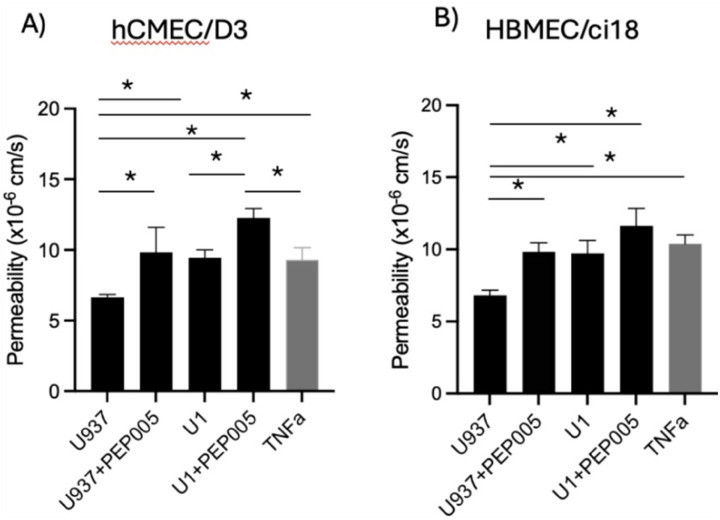
Effects of latent HIV conditioned media on BBB permeability. The endothelial cell lines hCMEC/D3 and HBMEC/ci18 were assembled with pericytes and astrocytes to test the effect of conditioned media from U937 (control) or U1 (latent) promonocytes, alone or after incubation with PEP005 10 nM, or media containing recombinant TNFa 10ng/ml as a positive control, on the permeability to Cascade Blue-labeled 10 kDa dextran particles. Data are expressed as mean of values obtained from at least 3 independent experiments ± SEM. *p<0.05 on indicated comparisons.

To validate the model, we compared the effects of the U1 with U937 conditioned media on the permeability to 10kDa dextran molecules in multicellular BBB systems containing either one of the cerebrovascular endothelial cells. U937-conditioned media did not affect BBB permeability significantly in neither one of the cell systems. In the presence of U1-conditioned media, significant disruption of the BBB integrity was observed in both hCMEC/D3 (Fig. 3A) and HBMEC/ci18 (Fig. 3B), as revealed by the increased permeability to dextran (Fig. 3), compared to the U937-conditioned media. The BBB disruption by U1 conditioned media occurred at similar levels to those triggered by TNFa as a positive control, which is also detected in the supernatants (Fig. 2). Importantly, the effect of conditioned media from cells stimulated with a latency reversal agent PEP005 showed increased BBB permeability regardless of virus in both cells. In hCMEC/D3 cells U1 + PEP005 increased permeability significantly above the positive control TNFa, but not in HBMEC/ci18. Yet, in relation to the > 1000-fold increase in viral transcription caused by PEP005 (Fig. 2A), the result suggests that virus in the supernatant had a rather modest or no contribution to the increase in BBB permeability. Thus, the exposure to U1 conditioned media was used as a model to mimic the BBB disruption in the context of latent HIV, such as in the CNS of PWH, and to test the effects of cannabinoids and their receptors in multicellular models bearing two cerebrovascular endothelial cell lines.

**Figure 4:**
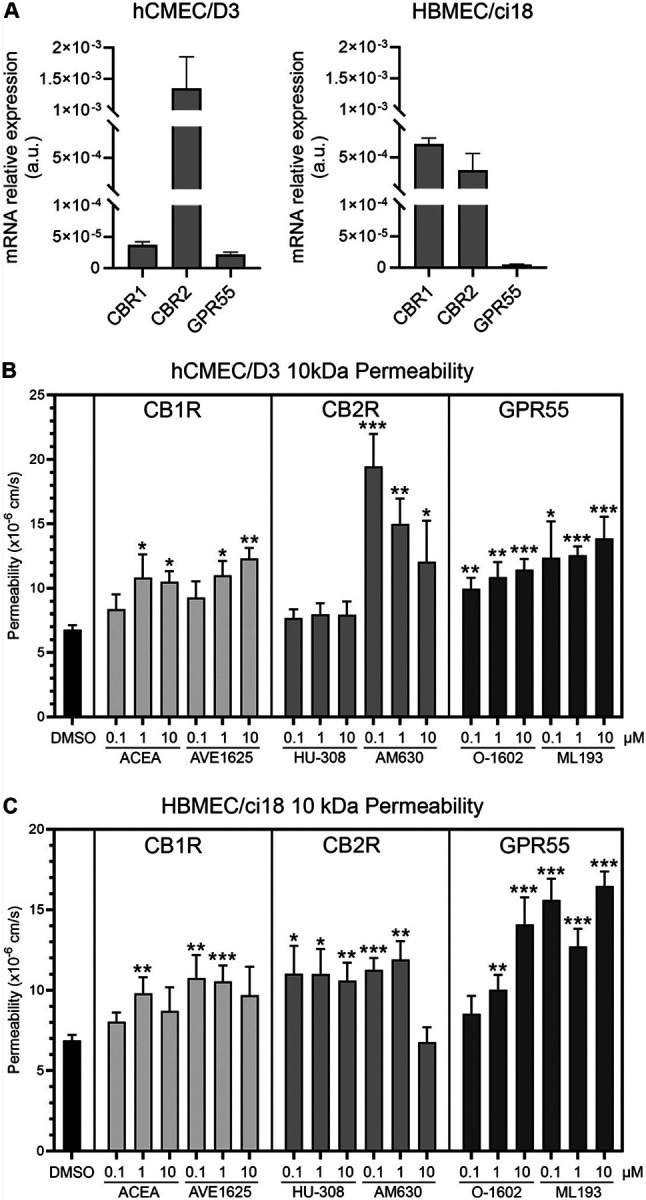
Endothelial cannabinoid receptors expression in hCMEC/D3 and HBMEC/ci18 cells and signaling effects on BBB permeability. **A)**Relative mRNA expression of CBR1, CBR2 and GPR55 in hCMEC/D3 and HBMEC/ci18 endothelial cells normalized to GAPDH. **B-C)** Permeability to dextran particles was tested in multicellular BBB systems in response to agonists and antagonists of indicated cannabinoid receptors. Effects of different doses of CBR agonists and antagonists on BBB permeability to 10 kDa dextran in hCMEC/D3 **(B)** and HBMEC/ci18 **(C).** DMSO was used as control for baseline effects. CB1R agonist ACEA and antagonist AVE1625, CB2R agonist HU-308 and antagonist AM630, and GPR55 agonist O-1602 and antagonist ML193 were provided at the indicated uM concentrations, for 24 hrs prior to the addition of dextran. Permeability was measured by fluorescence after 1 hr. Data are expressed as mean of values obtained from 3 independent experiments ± SEM. *p<0.05, **p<0.01, ***p<0.001 compared to DMSO controls.

First, we confirmed that the two endothelial cell lines express the major cannabinoid receptors CB1R, CB2R and GPR55 (Fig. 4A). Importantly, hCMEC/D3 cells had low transcriptional levels of CB1R, but high levels of CB2R. On the other hand, HBMEC/ci18 had higher CB1R expression. In these cells, CB2R was expressed, but at lower levels compared to hCMEC/D3 cells. Both cell lines had very low expression of GPR55. We then screened the ability of cannabinoid receptor signaling to modify the BBB disruption outcomes in both BBB systems. For that, we tested pharmacological agonists and antagonists to CB1R and CB2R, as well as GPR55 (Fig. 4B and 4C). The agonists and antagonists of CB1R and GPR55 all increased permeability of the BBB in both cell lines. On the other hand, the agonist for CB2R Hu308 stabilized the BBB in hCMEC/D3 cells, while its antagonist drastically increased permeability (Fig. 4B). However, this effect was not observed in HBMEC/ci18 cells in which permeability was increased with both CB2R agonist and antagonist (Fig. 4C). This result suggested that potential benefits to the BBB may result from CB2R signaling, facilitated by the agonist HU-308 in hCMEC/D3, and that the difference between these two endothelial cell lines may provide a potential mechanism for different effects observed in humans, and in the context of HIV.

Interestingly, incubation with CB1R agonist ACEA as well as the antagonist AVE1625, and with the GPR55 agonist O-1602 and antagonist ML-193 caused an increase in vascular permeability in both cell lines, suggesting that signaling through cannabinoid receptors other than CB2R on endothelial cells may enhance vascular injury indiscriminately (Figs. 4B and 4C).

**Figure 5:**
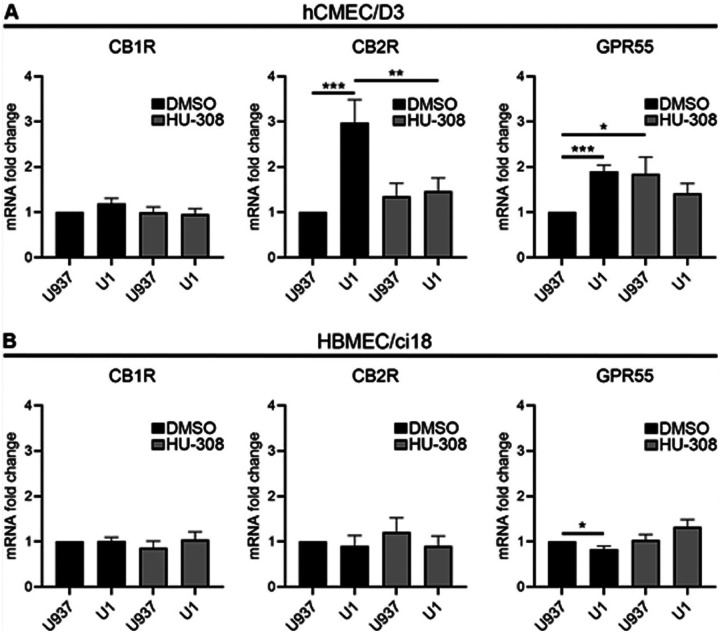
Latent HIV-infected environment and CB2R activation affect differently hCMEC/D3 and HBMEC/ci18 cannabinoid receptors expression. Fold change from mRNA baseline expression levels of CB1R, CB2R and GPR55 in **A)**hCMEC/D3 and **B)** HBMEC/ci18 after exposure to U937 or U1-conditioned medium, following a pre-incubation with DMSO or HU-308 (10 uM). Data are expressed as mean of values obtained from 3 independent experiments ± SEM. *p<0.05, ***p<0.001 for indicated comparisons.

We used the validated U1 conditioned media model to mimic HIV latent conditions to test whether the HIV latent environment characterized above modifies the levels of cannabinoid receptors on the endothelial cell lines and whether the CB2R agonist HU-308 modifies these effects (Fig. 5). In hCMEC/D3 cells, U1 conditioned media did not affect the expression of CB1R in the presence or absence of HU-308 but significantly increased the expression of CB2R and GPR55. The pretreatment with Hu308 partially prevented the change in levels of CB2R caused by U1-conditioned media. In addition, while HU-308 alone increased GPR55, the addition of U1 conditioned media to cells pretreated with HU-308 maintained GPR55 transcription at control levels (Fig. 5A). In HBMEC/ci18, neither U1 conditioned media nor HU-308 affected CB1R or CB2R expression. U1 conditioned media caused a modest but significant decrease of GPR55 transcription, which was prevented by the pretreatment with HU-308 (Fig. 5B). These data suggest that cerebral endothelial cells can differ in their expression of cannabinoid receptors, and that the environment containing HIV-latent myeloid cells modulates the levels of cannabinoid receptors differentially in the two endothelial cell lines. It also indicates that the HIV latent environment causes detrimental effects to endothelial health, and that signaling via CB2R can prevent these damaging effects in the cells that upregulate its transcription.

**Figure 6:**
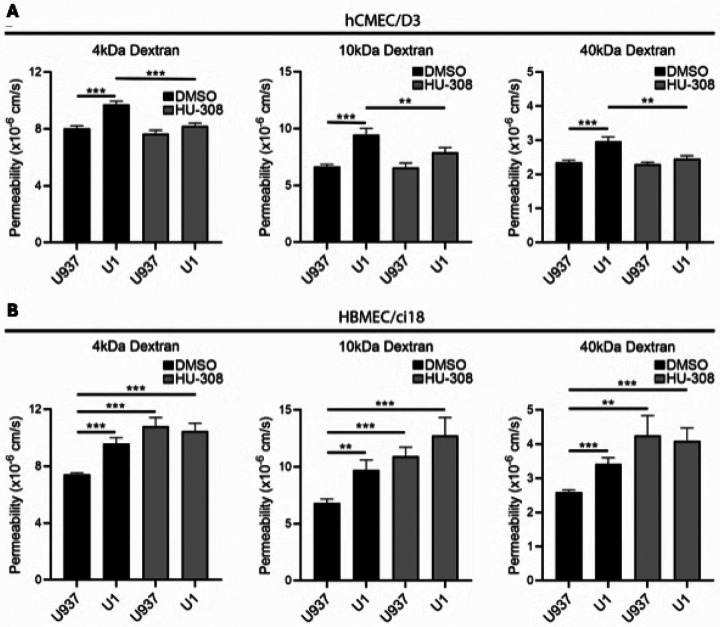
Effect of CB2R agonist HU-308 on hCMEC/D3- and HBMEC/ci18-containing BBB permeability. Endothelial cell permeability to 4, 10 and 40 kDa in multicellular BBB cultures containing **A)** hCMEC/D3 or **B)** HBMEC/ci18 endothelial cells, 24 hrs following the addition of control U937 conditioned medium or U1 latent HIV-conditioned medium, in presence of DMSO or 10 μM HU-308. Data are expressed as mean of values obtained from 3 independent experiments ± SEM. *p<0.05, **p<0.01, ***p<0.001 for indicated comparisons.

**Figure 7:**
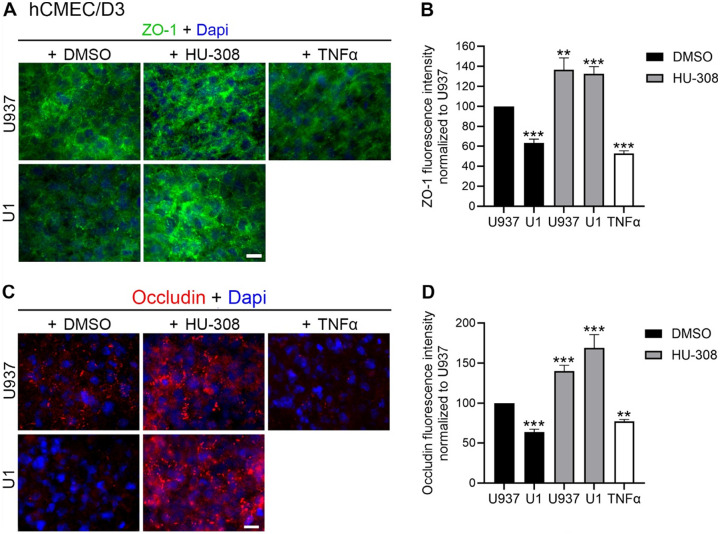
Effect of CB2R agonist HU-308 on the tight junction proteins ZO-1 and Occludin in BBB systems containing hCMEC/D3. Tight junction proteins were measured on hCMEC/D3 in multicellular BBB systems containing hCMEC/D3 endothelial cells, 24 hrs following the addition of control U937 conditioned medium or U1 latent HIV-conditioned medium, in presence of 0.1 % DMSO, 1 μM HU-308 or 10 ng/ml of TNFα (positive control for perturbation of the endothelial cell monolayer integrity). **A)** Representative images of ZO-1 expression (green) detected using immunohistochemistry on hCMEC/D3 endothelial cells in multicellular BBB cultures, and Dapi nuclear staining (blue) detected using immunohistochemistry. Scale bar = 10 μm. **B)** Quantification of ZO-1 fluorescence intensity by ImageJ/Fiji (NIH), normalized by area and to U937 control conditioned medium. Data are expressed as mean of 3 independent experiments in triplicate ± SEM.**p<0.01, ***p<0.001 compared to U937 untreated controls. **C)** Representative images of Occludin expression (red) and Dapi nuclear staining (blue) detected using immunohistochemistry on hCMEC/D3 endothelial cells in multicellular BBB cultures. **D)** Quantification of Occludin fluorescence intensity measured in ImageJ/Fiji (NIH), normalized by area and to U937 conditioned media as control. Data are expressed as mean of 3 independent experiments in triplicate ± SEM.**p<0.01, ***p<0.001 compared to U937 untreated controls.

Conversely, the stimulation of CB2R with HU-308 protected against the increase in permeability caused by U1 conditioned media in hCMEC/D3 as revealed by a reduction in permeability to all sizes of dextran particles (Figs. 6A), as well as by the enhanced expression of tight junction proteins ZO-1 (Figs. 7A and 7B) and occludin (Figs. 7C and 7D) measured by immunohistochemistry in these multicellular BBB systems.

**Figure 8:**
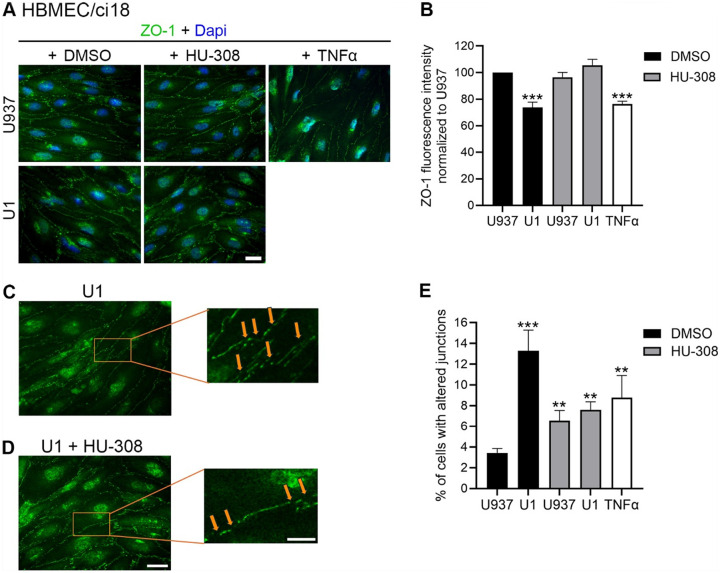
Effect of CB2R agonist HU-308 in ZO-1 expression pattern in BBB systems containing HBMEC/ci18. The tight junction protein ZO-1 was measured on HBMEC/ci18 endothelial cells in multicellular BBB systems. **A)** ZO-1 expression (green) was detected using immunohistochemistry in cultures exposed to U1 conditioned media for 24 h to control U937-conditioned medium or U1 latent HIV-conditioned medium, in presence of 0.1 % DMSO, 10 μM HU-308 or 10 ng/ml of TNFα. Dapi nuclear staining is shown in blue. Scale bar = 10 μm. **B)** Quantification of ZO-1 fluorescence intensity measured in ImageJ/Fiji (NIH), normalized by area and to U937 control conditioned medium. Data are expressed as mean of 3 independent experiments in triplicate ± SEM.**p<0.01, ***p<0.001 compared to U937 untreated controls. **C, D)** Magnification of representative images of HBMEC/ci18 membrane incubated with U1-conditioned media **C)** in the presence of DMSO control or **D)** in the presence of 1uM HU-308, allowing the observation of gaps in the expression of the tight junction protein (orange arrows). Scale bar, 5 μm. **E)** The number of cells showing membrane gaps on ZO-1 distribution was counted in each condition, following exposure to U937 or U1 conditioned media, following pretreatment with 10 uM HU-308 or the vehicle DMSO. TNFa was used as a positive control. **p<0.01, ***p<0.001 for indicated comparisons.

On the other hand, in HBMEC/ci18-containing BBB systems the addition of HU-308 alone or together with U1-conditioned media worsened BBB permeability (Fig. 6B), to all dextran sizes. Curiously, despite HU-308 worsened permeability in HBMEC/ci18 (Fig. 6B), it did prevent the loss of ZO-1 in those cells (Fig. 8A and 8B). However, the inspection of ZO-1 distribution patterns on the surface of HBMEC/ci18 cells indicated that HU-308-mediated recovery on ZO-1 levels only partially decreased gaps in tight junction expression caused by U1-conditioned media (Figs. 8C and 8D) confirmed by the quantification of the relative number of cells with membrane gaps (Fig. 8E).

**Figure 9:**
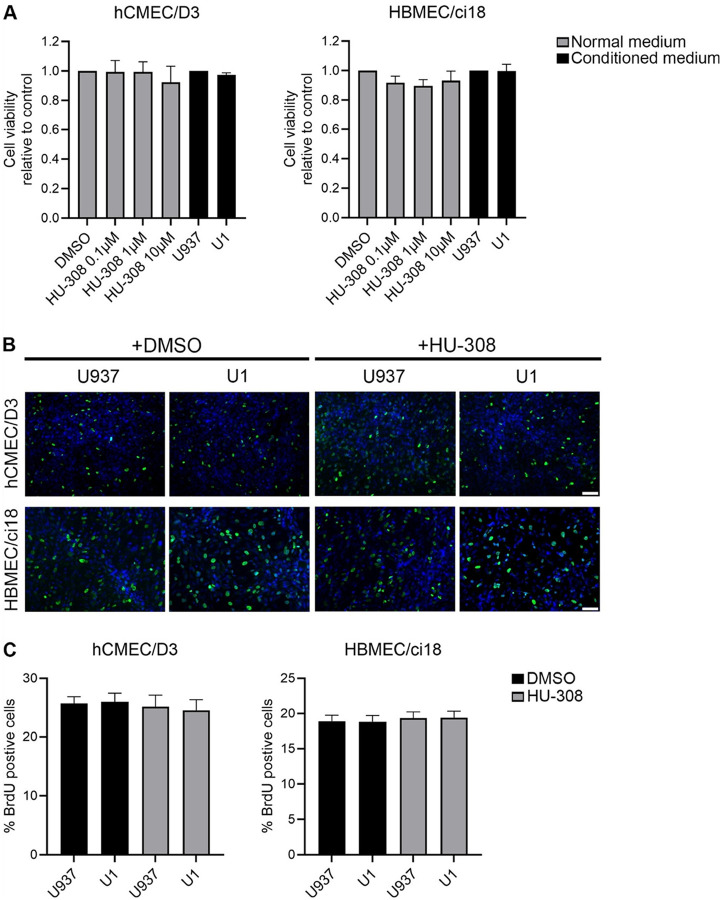
Cellular viability and proliferation of hCMEC/D3 and HBMEC/ci18 cells following exposure to promonocytes conditioned media and HU-308. **A)**Cell viability assay in hCMEC/D3 and HBMEC/ci18 measured by XTT after exposure to increasing concentrations of HU-308, together with control U937-conditioned media or U1- latent HIV-conditioned media. **B)** Representative images of BrdU incorporation (green) and Dapi nuclear staining (blue) in hCMEC/D3 and HBMEC/ci18 monolayers grown on glass coverslips for 5 days before being incubated for 24 h with control conditioned medium (U937) or latent HIV-conditioned medium latent (U1) in presence of 0.1 % DMSO or 10 μM HU-308 for 24 h. Scale bar = 50 μm. **C)** Quantification of the percentage of proliferating hCMEC/D3 and HBMEC/ci18 measured by BrdU incorporation after exposure to 10 uM HU-308, and conditioned media, using ImageJ/Fiji. Data are expressed as mean of values obtained from 3 independent experiments ± SEM. No statistical significance was identified.

As a potential explanation for these effects leading to hCMEC/D3 recovery and HBMEC/ci18 damage by HU-308, particularly in the context of HIV-latent conditioned media, we tested whether the CB2R agonist HU-308 affects the proliferation and survival of these cells (Fig. 9). However, neither the treatment with U1 conditioned media nor the pretreatment with HU-308 affected cell viability, as determined by XTT assay (Fig. 9A). Similarly, effects on proliferation determined by BrdU staining (Figs. 9B and 9C) were not observed.

**Figure 10:**
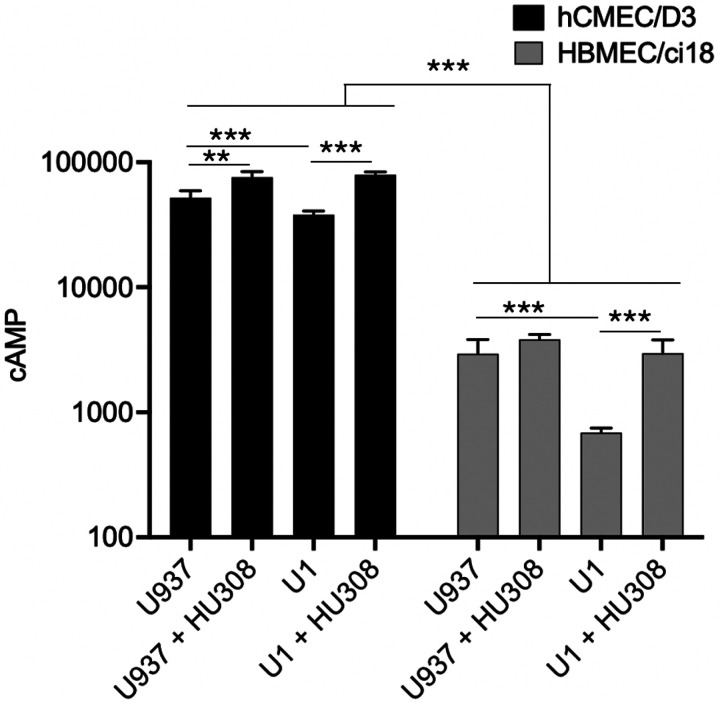
Detection of cyclic AMP in hCMEC/D3 or HBMEC/ci18 cells following U937 or U1-conditioned media, and effect of CB2R activation. The endothelial cultures were exposed to U937 or U1-conditioned media incubation, in the presence of DMSO or of 10 uM of HU-308. The cells were processed for the detection of cAMP measured by fluorescence and calculated with a standard curve. Data are expressed as mean of 3 independent experiments in triplicate ± SEM.**p<0.01, ***p<0.001 compared to U937 untreated controls.

To test whether differences in the signaling cascade triggered by the HU-308 binding to CB2R on hCMEC/D3 and HBMEC/ci18 may explain relative differences in the effects of the agonist, we measured cAMP downstream of the receptor activation (Fig. 10). Interestingly, we found a significant difference between the two cell lines in relation to the levels of cAMP, where hCMEC/D3 had 50–200-fold more cAMP at baseline compared to HBMEC/ci18 (Fig. 10). In hCMEC/D3, but not in HBMEC/ci18, HU-308 alone significantly increased cAMP levels. In both cell lines, U1-conditioned media significantly decreased cAMP to below baseline levels, and HU-308 promoted a recovery, suggesting that despite the drastically lower cAMP levels in HBMEC/ci18 compared to hCMEC/D3, the receptor that also occurs at lower levels in those cells (Fig. 4) is responsive to the agonist.

**Figure 11:**
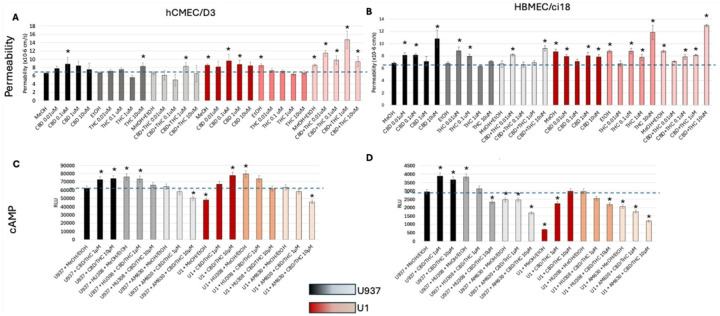
Effects of plant-derived cannabinoids on BBB systems’ permeability and on cyclic AMP induction in hCMEC/D3 or HBMEC/ci18 cells exposed to U937 or U1-conditioned media. **A-B)** Permeability to 10kDa dextran in multicellular BBB systems containing hCMEC/D3 cells **(A)** and HBMEC/ci18 cells **(B). C-D)** Normalized cAMP levels in single cultures of hCMEC/D3 **(C)** or HBMEC/ci18 **(D)** endothelial cells. Data are expressed as the mean of 3 independent experiments in triplicate ± SEM.**p<0.01, ***p<0.001 compared to U937 untreated controls.

To further confirm the differences between the two cell lines and validate the effects via CB2R, we treated the multicellular BBB cultures with plant-derived cannabinoids THC and CBD alone and combined. The cultures were tested for changes in the BBB integrity by measuring permeability to 10kDa dextran (Fig. 11A and B). Consistent with the experiments using the selective CB2R agonist HU-308, HBMEC/ci18 cells had more disruption of BBB integrity than hCMEC/D3 cells with both CBD or THC alone (Figs. 11A and 11B). Moreover, HBMEC/ci18 cells were not protected from the damaging effects of U1 conditioned media by THC or CBD (Fig. 11B), while in hCMEC/D3 cells pretreatment with THC prevented the increase in permeability that occurred after exposure to U1-conditioned media (Fig. 11A). However, CBD alone did not protect hCMEC/D3 cells against U1-latent media and even aggravated the increase in permeability when combined with THC (Fig. 11A).

Also, in agreement with the previous experiments, CBD and THC increased cAMP in both cell lines, in a CB2R-dependent manner, as indicated by the effects of the CB2R antagonist AM630, particularly when cannabinoids were added at higher doses (Figs. 11C and 11D). Yet, the levels of cAMP induced by the combined cannabinoids were up to 80-fold higher in hCMEC/D3 cells (Fig. 11C) compared to HBMEC/ci18 (Fig. 11D). Interestingly, the combination of HU-308 and THC/CBD in the context of U1-conditioned media decreased cAMP in a dose-dependent manner (Figs. 11C and 11D) in correlation with increased permeability in both cell lines (Fig. 11A). This suggests a potential reversal of the benefits via CB2R in pro-inflammatory conditions where other cannabinoid receptors are also activated. While cAMP is enhanced via selective CB2R activation, this result suggests that a signaling threshold is needed for beneficial effects and that the benefits may be overridden by the binding of cannabinoids to other receptors, masking or reversing benefits in the context of inflammation.

## Discussion

We used conditioned media from HIV-latently infected promonocytes to mimic the HIV- infected brain environment in the ART era, with low levels of virus and presence of low levels of pro-inflammatory and vasoactive components, yet able to cause a significant disruption of integrity in two BBB cell line systems, hCMEC/D3 cells[70, 71] and HMBEC/ci18 [72, 73]. Conditioned media from the uninfected parent promonocytes (U937) did not affect BBB integrity, validating the model to study BBB disruption in the context of HIV.

The loss of vascular integrity in both cell lines was characterized by decreased expression of TJ proteins such as ZO-1, and a significant increase in the permeability to dextran particles. Interestingly, the comparison of these two cell lines in response to the CB2R agonist Hu308 in the context of an HIV environment, indicated opposing effects. In hCMEC/D3, the CB2R agonist Hu308 prevented ZO-1 loss and decreased permeability to large molecules, suggesting it is beneficial to maintain BBB integrity. On the other hand, in HMBEC/ci18 cells Hu308 recovered ZO-1 expression to baseline levels but further increased permeability in all conditions due to an inability to redistribute tight junctions correctly. Interestingly, the beneficial versus damaging effects between the two cell lines aligned with significant differences in the levels of CB2R and the ability to build a sufficiently high cAMP storage. The decrease in cAMP caused by the HIV-environment was however corrected to baseline by the CB2R selective agonist in both cell types, and prevented by the selective antagonist, suggesting that the receptor is responsive in both cell lines, and that cAMP production is triggered via CB2R activation. Yet, the difference in baselines levels is revealing.

Whether residual viral particles in the latent cells-conditioned media can cause BBB disruption is an interesting question. Even upon the criticism that U1 cells do not transcribe productive virus, HIV peptides such as Tat can perturb endothelial health[74]. However, the drastic increase in p24 by latency reversal agents, such as PEP005, indicating viral transcription does not translate to proportional damage. Besides, the incubation of uninfected U937 promonocytes with the same latency reversal resulted in similar increase in BBB permeability. Since several studies have shown that cerebrovascular endothelial cells are strong gatekeepers and regulators of HIV infection, not likely permissive to infection, yet affected be HIV peptides such as Tat, Nef and gp120 and by chronic inflammation [75, 76], we deem that the damage to the BBB in the model is indirect, perhaps in part by residual viral peptides, but most likely due to the increased TNFa (used as a positive control for damage), along with other vasoactive cytokines.

The significance of the protective effects of CB2R expression and downstream signaling strength specifically in endothelial cells to promote vascular health suggests high clinical importance to identify individuals that will benefit from cannabinoids, and for explaining differences in the response among PWH[65]. The control of endothelial permeability by levels of cAMP has been previously reported, where higher levels are able to increase tight junction proteins[77]. Moreover, cAMP has been regarded as an endothelial barrier stabilizer in inflammatory conditions [78, 79]. The differences in cAMP levels between the two endothelial cell lines, both at baseline and elicited by CB2R explains different protective responses in the context of a latent HIV environment. One potential mechanism has been explored in hCMEC/D3 cells, where stimulation of adenosine receptors induces a Ca2 + influx by opening cyclic nucleotide-gated channels in a cAMP-dependent manner, causing formation and coupling of new gap junction plaques[80].

It is important to note that disturbing other cannabinoid receptors such as CB1R and GPR55 may lead to an increase in vascular permeability in both cell lines. The results of increased permeability in hCMEC/D3 cells stimulated with Hu308, THC and CBD simultaneously in the context of HIV latent supernatants indicates an interplay between these receptors, where excessive ligand availability and the binding to additional receptors may override the benefits of CB2R signaling, preventing recovery of cAMP levels that can be necessary to promote recovery. A decrease in net activation as a result of competitive binding of agonists and antagonists have been described in other GPCRs, either due to blocking or due to changes in receptor conformation[81, 82]. To date, more than 150 minor cannabinoids comprising less than 1% of the total dry mass of the plant, have been identified[83–85], indicating a complex pharmacology and potentially further increasing the range of responses in translational and real-world settings. Yet, the differential responses to CB2R and its activation dynamic in cerebrovascular endothelial cells, may explain previously observed differences in vascular markers in humans in the context of HIV compared to uninfected cannabis users [65], and depending on cannabis use patterns[58], which are predictive of neurocognitive outcomes. Our in vitro results performed in cerebrovascular endothelial cells suggest that levels of cAMP and activation thresholds may predict whether CB2R signaling will elicit beneficial outcomes culminating in recovery of the BBB integrity. The results also suggest that CB2R selective agonists may prove a more reliable way to deliver improvements in BBB function due to specificity. Another CB2R agonist, PM289, has been shown to improve barrier leakiness in a model using TNFa-induced damage in hCMEC/D3 cells in correlation with a moderation of the increase in NFkB availability [64].

This study has limitations due to the use of cell lines, which were favored here to minimize variability complicating the analysis of mechanisms. Other limitation is related to the emulation of the brain environment using conditioned media from latently infected cells. However, the rigorous proteome and viral characterization with the production of endothelial damage constituted a controlled model to screen the effects of cannabinoid compounds incorporating a diversity of models. In addition, the use of a well-characterized conditioned media was favored to eliminate the effects of cannabinoids on infected target cells in the system and allow isolation and control of BBB responses via targeted receptors. We have also not included antiretrovirals, which per se may contribute to BBB dysfunction despite keeping most drugs from entering the CNS[86], increasing the in vivo complexity. The complex pharmacological interactions between current HIV suppressive treatments and substance use are a separate chapter.

Using this controlled system with two different cerebrovascular cell lines, our results confirm the value of CB2R-mediated recovery of the BBB in the context of inflammation, regardless of latent virus, and suggest a contribution of a cAMP threshold underlying protective cellular events. While it is possible that upregulation of CB2R caused by inflammation enhances a signaling threshold that can shift the endothelial cell response via CB2R towards functional improvement, our results show that endothelial cells stimulated with HIV-latent U1-derived conditioned media had a proportional decrease in levels cAMP, recovered by the selective agonist and more likely by THC. However, the overall higher expression of CB2R in hCMEC/D3 compared to HBMEC/ci18, leading to beneficial conditions, may contribute to an increased strength of activation of this signaling pathway, thereby promoting vascular health.

## Data Availability

The datasets used and/or analysed during the current study are available from the corresponding author on reasonable request.

## Abbreviations

BBB: Blood-Brain Barrier
BrdU: 5-Bromo-2’-deoxy-Uridine
BSA: Bovine Serum Albumin
cAMP: cyclic adenosine 3,5 monophosphate
CB1R: Cannabinoid Receptor-1
CB2R: Cannabinoid Receptor-2
CBD: Cannabidiol
CBRs: Cannabinoid Receptors
CMEC: cerebral microvascular endothelial cells
CNS: Central Nervous System
CSF: Cerebrospinal Fluid
CST3: Cystatin C
CVD: Cardiovascular Didease
DAPI: 4′,6-diamidino-2-phenylindole
DMEM: Dulbecco’s Modified Eagle Medium
DMSO: Dimethyl sulphoxide
ECS: Endocannabinoid system
EGF: Endothelial Growth factor
ELISA: Enzyme-linked Immunosorbent Assay
FAAH: Fatty Acid Amide Hydrolase
GAPDH: Glyceraldehyde-3-phosphate dehydrogenase
GFAP: Glial Fibrillary Acidic Protein
GPR55: G-protein receptor 55
GS: Glutamine Synthetase
HASTR: human astrocytes
HBMEC: human brain microvascular endothelial cells
HBPC: human brain pericytes
HBSS: Hank’s balanced salt solution
HIV: Human Immunodeficiency Virus
ICAM-1: intercellular adhesion molecule - 1
IGFBP4: insulin-like growth factor binding protein-4
MAGL: Monoacylglycerol lipase
MAPK: mitogen-activated protein kinase
MPO: myeloperoxidase
NCI: Neurocognitive Impairment
PBS: calcium- and magnesium-free phosphate buffered saline
PET: clear polyester
PI3K: activate phosphoinositide 3-kinase
PIGF: Placental Growth Factor
PMA: Phorbol Miristate Acetate
PWH: Persons living with HIV
RAGE: receptor for advanced glycation end products
RNA: Ribonucleic Acid
SAA: Serum Amyloid A
SPP1: Osteopontin
THC: Δ9-tetrahydrocannabinol
TJ: tight junctions
TNFa: Tumor Necrosis Factor-alpha
uPAR: urokinase activator plasminogen receptor
VCAM-1: vascular cell adhesion molecule - 1
VEGF: Vascular Endothelial Growth Factor
ZO-1: Zonula Occludens-1
